# Prion Protein Protects Cancer Cells against Endoplasmic Reticulum Stress Induced Apoptosis

**DOI:** 10.1007/s12250-019-00107-2

**Published:** 2019-04-24

**Authors:** Zhenxing Gao, Min Peng, Liang Chen, Xiaowen Yang, Huan Li, Run Shi, Guiru Wu, Lili Cai, Qibin Song, Chaoyang Li

**Affiliations:** 1 State Key Laboratory of Virology, Wuhan Institute of Virology, Chinese Academy of Sciences, Wuhan, 430071 China; 2 Department of Oncology, Renmin Hospital of Wuhan University, Wuhan, 430060 China; 3 Department of the First Abdominal Surgery, Jiangxi Tumor Hospital, Nanchang, 330029 China

**Keywords:** Endoplasmic reticulum stress, Brefeldin A (BFA), Prion protein (PrP), Glycosylation, Apoptosis

## Abstract

Unfolded protein response (UPR) is an adaptive reaction for cells to reduce endoplasmic reticulum (ER) stress. In many types of cancers, such as lung cancer and pancreatic cancer, cancer cells may harness ER stress to facilitate their survival and growth. Prion protein (PrP) is a glycosylated cell surface protein that has been shown to be up-regulated in many cancer cells. Since PrP is a protein prone to misfolding, ER stress can result in under-glycosylated PrP, which in turn may activate ER stress. To assess whether ER stress leads to the production of under-glycosylated PrP and whether under-glycosylated PrP may contribute to ER stress thus leading to cancer cell apoptosis, we treated different cancer cells with brefeldin A (BFA), thapsigargin (Thps), and tunicamycin (TM). We found that although BFA, Thps, and TM treatment activated UPR, only ATF4 was consistently activated by these reagents, but not other branches of ER stress. However, the canonical PERK-eIF2α-ATF4 did not account for the observed activation of ATF4 in lung cancer cells. In addition, BFA, but neither Thps nor TM, significantly stimulated the expression of cytosolic PrP. Finally, we found that the levels of PrP contributed to anti-apoptosis activity of BFA-induced cancer cell death. Thus, the pathway of BFA-induced persistent ER stress may be targeted for lung and pancreatic cancer treatment.

**Electronic supplementary material:**

The online version of this article (10.1007/s12250-019-00107-2) contains supplementary material, which is available to authorized users.

## Introduction

Lung cancer and pancreatic ductal adenocarcinoma (PDAC) are two of the most deadly malignant tumors worldwide (Ferlay *et al*. 2013; Siegel *et al.*^2016^). The five-year survival rate was 6% for both tumors owing to the lack of diagnostic markers or appropriate screening tests, and drug resistance to cancer treatment (Jemal *et al.*^2008^; Maisonneuve and Lowenfels 2010). Most importantly, cancer burden including lung cancer and PDAC in China will continue to increase in the next decades (Chen *et al.*^2016^). Thus, understanding how cellular factors contribute to the tumorigenesis of lung cancer and PDAC is very important for the development of a treatment approach.

Cellular prion protein (PrP) is a glycosylphosphatidylinositol (GPI)—anchored protein highly expressed in neuron cells of the central nervous system. PrP has been attributed to cell adhesion, anti-apoptosis, migration, signaling, viral replication, immune modulation, and cell differentiation (Brown *et al.*^1997^; Kuwahara *et al.*^1999^; Mouillet-Richard *et al.*^2000^; Bounhar *et al.*^2001^; Paitel *et al.*^2004^; Wu *et al.*^2017^; Zhang *et al.*^2017^). However, the physiological function of this protein remains elusive, although it is known to be essential for transmissible spongiform encephalopathy (Prusiner 1998). The expression of PrP is undetectable in normal pancreatic ductal cells or hepatocytes (Bendheim *et al.*^1992^; Li *et al.*^2009^; Yang *et al.*2014a). However, in some solid tumors, such as PDAC, oral squamous cell carcinoma, colon cancer, breast cancer, gastric cancer, melanoma, and glioma, the expression level of PrP is up-regulated (Yang *et al.*2014b). More importantly, expression of PrP has been reported as a biomarker for poor prognosis for PDAC, breast cancer, and gastric cancer (Li *et al.*^2009^; Dery *et al.*^2013^; Zhou *et al.*^2014^).

Accumulation of misfolded or unfolded proteins in the endoplasmic reticulum (ER) causes ER stress, which may activate an unfolded protein response (UPR). At least three sensors of ER stress have been identified: (1) inositol-requiring protein 1 (IRE1); (2) pancreatic-like ER kinase like- kinase (PERK); and (3) activating transcription factor 6 (ATF6). IRE1α is activated upon dimerization to produce an active transcription factor, spliced X-box binding protein 1 (XBP-1s), which controls, for example, the transcription of gene encoding proteins for ER-associated degradation (ERAD) and phospholipid synthesis (Rao and Bredesen 2004). PERK phosphorylates the initiation factor eukaryotic translation initiator factor 2α (eIF2α), nuclear factor erythroid 2-related factor 2 (NRF2), forkhead box O, and diacylglycerol (Shi *et al.*^1998^; Harding *et al.*^1999^; Cullinan and Diehl 2004; Bobrovnikova-Marjon *et al.*^2012^; Zhang *et al.*^2013^). Phosphorylated eIF2α may then result in the modulation of autophagy, apoptosis, amino acid metabolism, or antioxidant responses. Under ER stress, ATF6 binds coat protein II complex to enter the Golgi apparatus where it is cleaved by site 1 protease (S1P) and site 2 protease (S2P) to release the cytosolic domain fragment ATF6f (Ye *et al.*^2000^). ATF6f further fine tunes the genes involved in ERAD and XBP-1.

Several chemicals, such as tunicamycin (TM), thapsigargin (Thps), and brefeldin A (BFA) have been used extensively to induce ER stress in cell models. These agents appear to induce UPR via different mechanisms as TM is a potent inhibitor of GlcNAc phosphotransferase to prevent glycosylation of glycoprotein; Thps depletes calcium in ER; whereas BFA impedes protein transport from the ER to the Golgi apparatus (Takatsuki Akira and Gakuzo 1975; Misumi *et al.*^1986^; Thastrup *et al.*^1990^).

Due to their increased metabolic requirements, tumor cells engage UPR to promote their growth demand, survival, and metastasis (Corazzari *et al.*^2017^). Lung cancer and PDAC cells are highly secretory and prone to constitutive UPR activation and thus may harness ER stress for their own privilege (Lee and Hendershot 2006; Wang and Kaufman 2014). However, the relationship between PrP expression and ER stress in cancer cells remains obscure. In HeLa and N2a cells, ER stress reduces total PrP level (Orsi *et al.*^2006^; Nunziante *et al.*^2011^). On the contrary, up-regulation of PrP was observed with ER stress in breast cancer (Dery *et al.*^2013^). Therefore, we hypothesize that the interplay between PrP and ER stress may be cell-context dependent.

PrP is known to be important in tumorigenesis, therefore we aimed to investigate the role PrP plays in cancer cells during ER stress to better understand the action of two of the deadliest cancers worldwide, lung cancer and PDAC.

## Materials and Methods

### Cell Lines

PDAC cell line BxPC-3 was purchased from American Type Culture Collection (ATCC). *PRNP* null BxPC-3 cells were generated as previously described (Yang *et al.*^2016^). Non-small-cell lung carcinoma cell lines A549, H157, H1299, and SPC-A1 were obtained from China Center for Type Culture Collection. These cells were cultured in RPMI1640 medium (31800-022, Gibco, Grand Island, NY, USA) supplemented with 10% FBS (10099-141, Gibco), 1.5 g/L sodium bicarbonate, 4.5 g/L glucose, 3 g/L HEPES (V900477, Sigma-Aldrich, Darmstadt, Germany), 1 mmol/L sodium pyruvate (11360-070, Gibco), 100 U/mL of antibiotic penicillin–streptomycin solution (03-031-1, Biological Industries, Kibbutz Beit-Haemek, Israel) in a humidified atmosphere containing 5% CO_2_.

### Antibodies and Reagents

Anti-PrP specific monoclonal antibody (4H2) was generated in our laboratory as previously described (Yang *et al.*2014a). Anti-β-actin mouse monoclonal antibody (KM9001) was purchased from Tianjin Sungene Biotech (Tianjin, China). PARP-1 antibody (9542), ATF4 antibody (11815), phospho-eIF2α (p-eIF2α, 3398), eIF2α (5324), p53 (9282), caspase-3 (9665), and cleaved caspase-3 (9661) were purchased from Cell Signaling Technology (Danvers, MA, USA). Glucose response protein 78 or binding immunoglobulin protein (Grp78/BiP) antibody (11587-1-AP) was purchased from Proteintech (Wuhan, Hubei, China). Anti-XBP-1 antibody (ab37152) was purchased from Abcam (Cambridge, MA, USA). Horseradish peroxidase (HRP)-conjugated goat anti-mouse IgG (H+L) antibody (AS003) and HRP-conjugated goat anti-rabbit IgG antibody (AS014) were purchased from Abclonal (Wuhan, China). Alexa Fluor 488 conjugated goat anti-rabbit IgG (H+L) secondary antibody (R37116) and Alexa Fluor 555 conjugated goat anti-mouse IgG (H+L) secondary antibody (A32727) were purchased from Invitrogen (Eugene, OR, USA). 4′, 6-diamidine-2′-phenylindole dihydrochloride (DAPI) (10236276001) was purchased from Roche (Mannheim, Germany). BFA (S1536) was purchased from Beyotime (Shanghai, China). Dimethyl sulfoxide (DMSO, D2650), Thps (T9033) and TM (T7765) were purchased from Sigma-Aldrich. Other reagents reported in the paper were purchased from Amresco (Solon, Ohio, USA). All reagents purchased from commercial sources were used according to the suppliers’ recommendations.

### Cell Lysate Preparation

Cells were seeded in 12-well plates overnight. When cell confluence reached 70%–80%, the medium was changed with fresh medium supplemented with the indicated concentrations of BFA, Thps and TM. Dimethyl sulfoxide (DMSO) was used as vehicle control. After 24 h, the cells were rinsed twice with ice cold phosphate buffered saline (PBS). Cell lysate was made in cell lysis buffer (20 mmol/L Tris–HCl (pH 7.5), 150 mmol/L NaCl, 1 mmol/L EDTA, 1 mmol/L EGTA, 1% Triton X-100, 2.5 mmol/L sodium pyrophosphate, 1 mmol/L β-glycerol phosphate, 1 mmol/L Na_3_VO_4_); and 1 mmol/L PMSF and protease inhibitor cocktail (04693116001, Roche) were added freshly. Protein concentration was determined by Bio-Rad Protein Assay Kit II (5000002, Hercules, CA, USA).

### Peptide-N-Glycosidase F (PNGase F) Treatment

An amount of 20 μg of proteins per sample was combined with 1 μL 10 × glycoprotein denaturing buffer and deionized water to make a 10 μL reaction volume on ice. The sample was then boiled for 10 min. Then, 2 μL 10 × G7 buffer, 2 μL 10% NP40, 5 μL deionized water and 1 μL PNGase F (P0704, New England Biolabs, Ipswich, MA, USA) were added to make a total of 20 μL reaction mixture. PNGase F treated samples were then subjected for immunoblotting.

### Immunoblotting

Samples were mixed with 4 × sample reducing buffer (40% glycerol (V/V), 250 mmol/L Tris-HCl pH 6.8 (V/V), 8% sodium dodecyl sulfate (SDS, W/V), 0.04% bromophenol blue (W/V), and 20% β-mercaptoethanol (V/V)). The samples were then heated at 100 ºC in a heat block for 5 min. Samples were separated on a 10% SDS–polyacrylamide gel electrophoresis (PAGE) gel, and then transferred to nitrocellulose membrane. After blocking in 3% bovine serum albumin (BSA) in TBST (137 mmol/L NaCl, 20 mmol/L Tris, 0.1% Tween 20, pH7.6), the blots were probed by the indicated primary antibodies at the indicated concentrations. Bound primary antibody was further probed with HRP-conjugated goat anti-mouse IgG secondary antibody (1:10,000) or HRP-conjugated goat anti-rabbit IgG secondary antibody (1:10,000).

### RNA Isolation and Quantitative PCR

Total RNA was isolated from cultured cells using an RNA purification kit (TR01-150, GeneMark, Taichung, Taiwan). An amount of 0.8 μg RNA was reverse transcribed to cDNA by using a PrimeScriptTM RT reagent kit (RR047A, TaKaRa, Shiga, Japan). Quantitative PCR (qPCR) was carried out on a Bio-Rad real-time PCR instrument (CFX Connect Real-Time PCR Detection System, Bio-Rad). Each reaction volume of 20 μL contained cDNA templates, primer pairs, and SYBR Green Supermix (170-8882AP, Bio-Rad). Amplification occurred after initial denaturation at 95 °C for 3 min, followed by 40 cycles of 95 °C for 15 s, 60 °C for 30 s, and 72 °C for 20 s. *β*-*ACTIN* was used as a reference gene. Gene-specific primers used for qPCR are listed in Supplementary Table S1.

### Immunofluorescence Staining

Cells were seeded into poly-d-lysine-coated glass bottom petri dishes overnight. To detect the cell surface PrP, cells were washed with ice cold PBS three times then the cells were incubated with 5 µg/mL 4H2 or isotype control mouse IgG1 for 1 h at room temperature. Bound antibodies were probed with AlexaFluor 555 conjugated goat anti-mouse IgG. DAPI was used to counterstain the nuclei. Images were taken with Olympus inverted microscopy (Tokyo, Japan). To detect the co-immunostaining of PrP and BiP, cells were prepared as above. After 24 h BFA treatment, the cells were washed with ice cold PBS three times. Cells were then fixed in 4% paraformaldehyde for 15 min at room temperature and washed with PBS three times. After blocking for 1 h (1% BSA, 10% goat serum diluted in PBSTT (0.1% tween 20, 0.3% Triton X-100)), 4H2 (10 µg/mL) and BiP (1:100) antibody in blocking buffer were applied for 1 h. Bound antibodies were probed with Alexa Fluor 555 conjugated goat anti-mouse IgG and Alexa Fluor 488 conjugated goat anti-rabbit IgG. Images were taken with A1 MP+ multiphoton confocal microscope (IMA101065ALS, Nikon, Japan) after being counterstained with DAPI and immersed with antifade.

To detect cell apoptosis, we used an Annexin V-FITC cell apoptosis kit (C1062, Beyotime) for in situ immunostaining and flow cytometry analysis. Briefly, for *in situ* immunostaining, cells were seeded in 12-well plates overnight. After 16 h, the medium were changed with fresh medium supplemented with the indicated concentrations of BFA or DMSO for an additional 24 h. Cells were then washed twice with ice cold PBS, and then incubated with 210 μL of apoptosis detection buffer (195 μL Annexin V-FITC binding buffer, 5 μL Annexin V-FITC, 10 μL propidium iodide) for 15 min at room temperature in the dark. Images were taken with Olympus inverted microscopy.

### Flow Cytometry Analysis

To quantify cell apoptosis with flow cytometry, cells were seeded in 6-well plates overnight. After 16 h, the medium were replaced with fresh medium supplemented with indicated concentration of BFA or DMSO for an additional 24 h. The cells were then scraped and digested with trypsin/EDTA. Digested cells were centrifuged at 1000 ×*g* for 5 min at 4 ºC. After washing with PBS once, cells were transferred in a 1.5 mL tube and were further stained with 5 μL of Annexin V-FITC and 10 μL of PI in 195 μL Annexin V-FITC binding buffer for 15 min at room temperature in the dark. The samples were analyzed in a FACS AriaIII flow cytometer (BD Biosciences, NJ, USA).

### Statistical Analysis

Data are expressed as mean ± SEM (standard error of the mean). Statistical analysis was performed using 2-tailed student’s *t* test. A value of *P *< 0.05 was considered statistically significant. The following levels of statistical significance were used: *P < 0.05; **P < 0.01; ***P < 0.001.

## Results

### Some Lung Cancer Cell Lines Express PrP

**Fig. 1:**
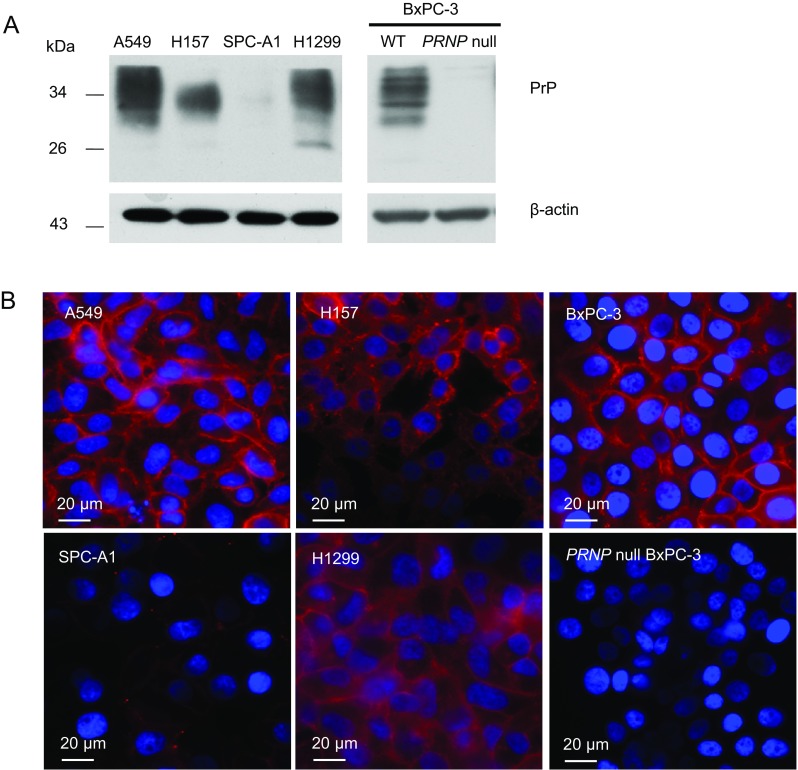
Cancer cells express different levels of PrP. **A** Immunoblotting with PrP specific mAb 4H2 showed that A549, H157, H1299, and BxPC-3 cells expressed PrP. A residual level of PrP was detected in SPC-A1 cell lysate. *PRNP* null BxPC-3 cells were used as negative control. β-actin was used a loading control. **B** Confocal immunofluorescence staining with 4H2 revealed that A549, H157, H1299, and BxPC-3 cells expressed PrP, and most PrP was cell surface bound. On the contrary, no signal of PrP was detected in SPC-A1 and *PRNP* null BxPC-3 cells. Nuclei were counter stained with DAPI. The experiments were repeated three times with similar results.

PrP is expressed at low levels in normal human lung tissue (The Human Protein Atlas). Whether lung cancer cell lines express PrP has never been studied in detail. Therefore, to investigate if lung cancer cell lines express PrP, we blotted cell lysates from A549, H157, SPC-A1, H1299, and BxPC-3 cells with PrP-specific mAb 4H2 and found that A549, H157, H1299 and BxPC-3 cells expressed PrP, while only SPC-A1 did not (Fig. 1A). Confocal immunofluorescence staining confirmed that A549, H157, H1299, and BxPC-3 cells expressed PrP and most of the PrP was cell surface bound (Fig. 1B). Therefore, most lung cancer cell lines express PrP.

### ER Stress Induces Activating Transcription Factor 4 (ATF4) Expression

**Fig. 2:**
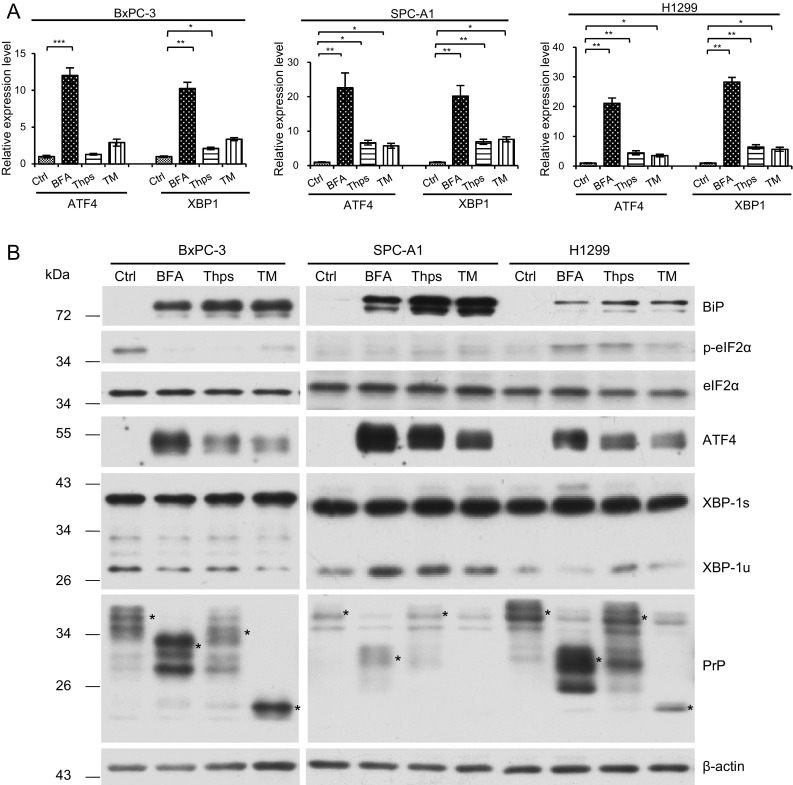
ER stress enhances *ATF4* and *XBP*-*1* mRNA levels in BxPC-3, SPC-A1 and H1299 cells. **A** ER stress was induced by culturing cells in the presence of BFA, Thps, or TM for 24 h. DMSO was used as vehicle control (Ctrl). Expression of *ATF4* and *XBP*-*1* mRNA levels was quantified by qPCR and normalized by *β*-*ACTIN*. BFA, Thps, or TM activated significant expression of *ATF4* and *XBP*-*1* transcripts in SPC-A1 and H1299 cells. However, only BFA induced significant *ATF4* expression in BxPC-3 cells. In addition, significant expression of *XBP*-*1* was induced by BFA and Thps in BxPC-3 cells. Statistical significance was indicated as: *P < 0.05; **P < 0.01; ***P < 0.001. **B** BFA activates PrP expression via ER stress. BFA, Thps, and TM induced expression of BiP. BFA reduced p-eIF2α expression in BxPC-1 cells but not in SPC-A1 and H1299 cells. However, in all tested cells, eIF2α levels were not altered by the treatment. BFA, Thps, and TM activated ATF4 levels in all tested cancer cells. *XBP*-*1* was constitutively activated in all tested cancer cells. Increased PrP levels were detected in all tested cancer cells by BFA but neither Thps nor TM treatment. PrP was indicated by *. Some proteins reacting to 4H2 were non-specific. The experiments were repeated three times.

The promoter region of PrP has been reported to have XBP-1, ATF4, and ATF6 binding elements. Treatment of breast cancer cell lines with chemicals inducing ER stress results in PrP expression (Dery *et al.*^2013^). To investigate if BFA, Thps, and TM treatment of BxPC-3, SPC-A1, and H1299 up-regulated UPR response, we treated these cells *in vitro* with BFA, Thps, or TM for 24 h. We found that BFA treatment of BxPC-3, SPC-A1, and H1299 cells significantly enhanced mRNA levels of *ATF4* and *XBP*-*1* (Fig. 2A). In addition, Thps and TM also enhanced mRNA of *ATF4* and *XBP*-*1* in SPC-A1 and H1299 cells but at significantly lower levels compared to the effects of BFA (Fig. 2A). On the contrary, Thps, but not TM, activated *XBP*-*1* mRNA level in BxPC-3 cells (Fig. 2A). These results imply that transcriptional factors such as *ATF4* and *XBP*-*1* may be activated in cancer cells by BFA.

To confirm the mRNA findings, we blotted those cell lysates with antibodies specific for p-eIF2α, total eIF2α, ATF4, and XBP-1, respectively, to identify which pathway(s) was activated. P-eIF2α has a translational inhibition effect on most proteins; however, it enhances the translation of ATF4. We found that in BxPC-3 cells, BFA, Thps, and TM treatment reduced the level of p-eIF2α (Fig. 2B). However, in H1299 cells, BFA, Thps, and TM treatment increased p-eIF2α level, whereas p-eIF2α was not detected in SPC-A1 cells (Fig. 2B). In contrast to p-eIF2α, when cell lysates were blotted with antibody specific for ATF4, we found that ATF4 was significantly up-regulated at 24 h post BFA, Thps, and TM treatment, although Thps and TM were not as efficient as BFA to activate ATF4 in those cells (Fig. 2B). Therefore the canonical PERK-eIF2α-ATF4 might occur only in H1299 cells but not in BxPC-3 and SPC-A1 cells.

Activated XBP-1 (XBP-1s) has an apparent molecular weight of 40 kDa, whereas the non-spliced form of XBP-1 (XBP-1u) is approximately 29 kDa. When blotted with anti-XBP-1 specific antibody, we found that all tested cancer cells expressed an active form of XBP-1s, even in the absence of BFA, Thps, and TM treatment (Fig. 2B). However, lung cancer cells SPC-A1 and H1299 expressed higher levels of XBP-1s than PDAC cells BxPC-3. These results suggested that XBP-1s is constitutively activated in those cancer cells.

Since up-regulation of BiP could be a marker for UPR (Kaufman 1999), we blotted the cell lysates from treated cells with anti-BiP specific antibody to investigate whether UPR occurred in those cells. We found that all treated cells showed significantly enhanced BiP levels (Fig. 2B). These results suggest that indeed UPR was induced in those cells by different reagents, albeit at different levels. Interestingly, when low-expressing PrP SPC-A1 cells were treated with UPR stimulants, they expressed higher levels of both ATF4 and BiP compared to high-expressing PrP H1299 and BxPC-3 cells which were treated the same way. Therefore, cells with lower PrP levels might respond better to ER stress than cells with higher PrP levels. In other words, PrP is a negative regulator of ER stress.

### BFA Activated ER Stress Induces PrP Expression

**Fig. 3:**
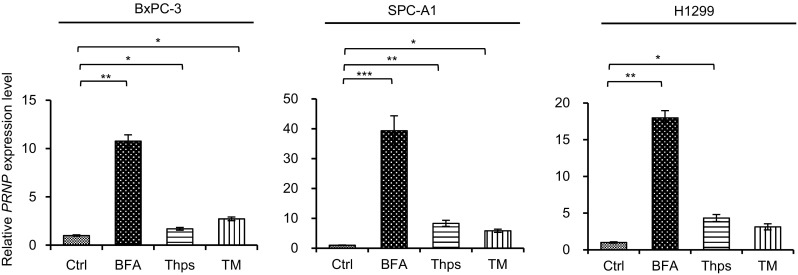
*PRNP* transcripts were significantly elevated in SPC-A1 and BxPC-3 cells by BFA treatment. BFA, Thps, and TM treatment significantly enhanced *PRNP* levels in BxPC-3 and SPC-A1 cells. However, BFA and Thps but not TM significantly activated *PRNP* expression in H1299 cells. DMSO was used as vehicle control (Ctrl). The experiments were repeated three times. Statistical analyses were indicated as: *P < 0.05; **P < 0.01; ***P < 0.001.

We then investigated whether ER stress induced PrP expression. We first blotted cell lysates with 4H2 and found significant up-regulation of PrP at 24 h after BFA treatment for all tested cells (Fig. 2B, indicated by *). Similarly, significantly more PrP expression was observed in H1299 cells than in SPC-A1 cells (Fig. 2B, indicated by *). In contrast to BFA treatment, Thps and TM treatment did not induce, and in some cases reduced PrP expression at protein levels in these cancer cells (Fig. 2B). More importantly, the expression pattern of PrP was different in BxPC-3 cells as compared to PrP in lung cancer cells (Fig. 2B). The reason for this difference is unknown. To further confirm that the up-regulated expression of PrP is due to ER stress, which stimulated the ATF4 transcriptional factor to transcribe *PRNP*, we performed qPCR to quantify *PRNP* mRNA levels after treatment. In BxPC-3 and SPC-A1 cells, BFA, Thps, and TM activated significant *PRNP* transcription. In contrast, in H1299 cells, BFA and Thps, but not TM, treatment activated significant *PRNP* transcripts (Fig. 3). Thus, BFA and Thps seemed to be able to induce *PRNP* transcription via ER stress in all tested cells.

### PrP Expression is Not Enhanced by p53 when Treated with BFA

**Fig. 4:**
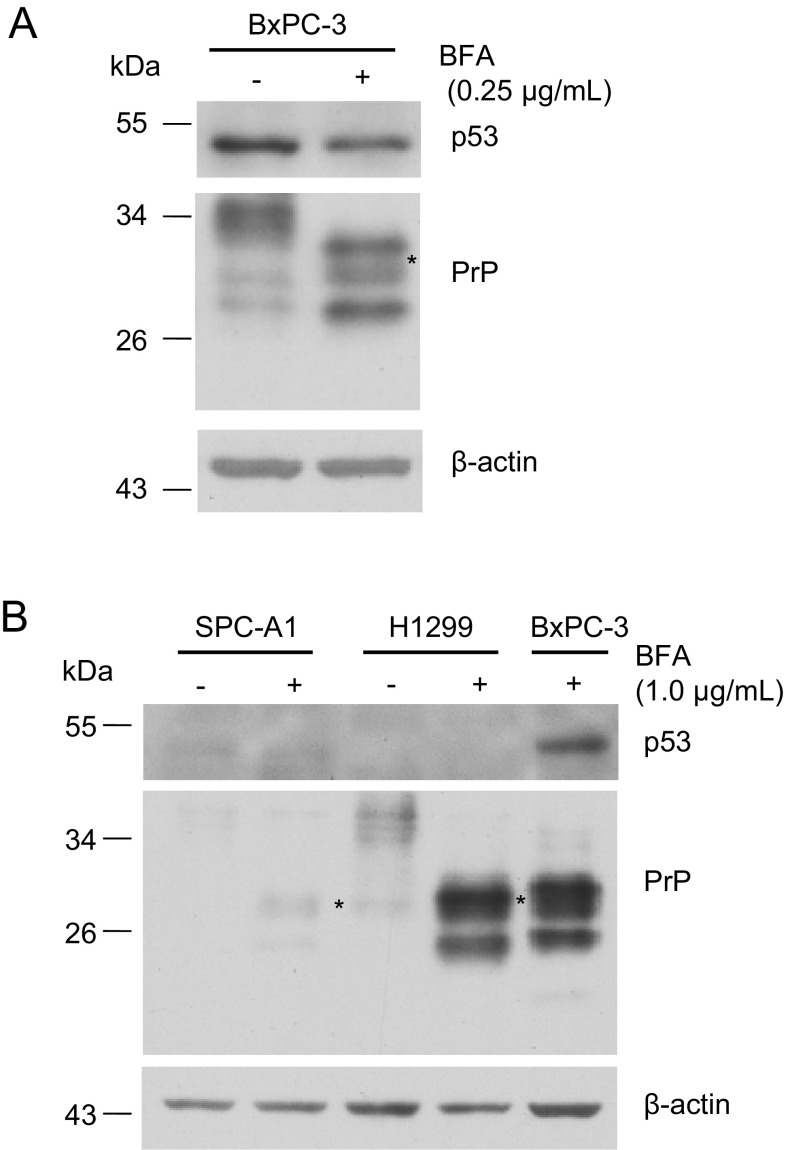
p53 is not the transcript factor for PrP under BFA-induced ER stress. **A** PrP expression is not correlated with p53 in PDAC cells. PrP expression is up-regulated in BxPC-3 cells under BFA treatment. However, reduced p53 was detected in BxPC-3 cells treated with BFA. **B** PrP expression is not correlated with p53 in lung cancer cells. Slight up-regulation of PrP (indicated with *) or significant up-regulation of PrP was detected in SPC-A1 and H1299 cells, respectively when these cells were treated with BFA. Residual p53 or no p53 was detected in these two cancer cell lines. As a positive control, p53 was detected in BxPC-3 cells treated with BFA.

In addition to motifs binding XBP-1, ATF4, and ATF6, the promoter region of *PRNP* contains the motif for p53 binding (Vincent *et al.*^2009^). To exclude the possibility that BFA-induced PrP expression was transcribed by p53, we treated BxPC-3, H1299, and SPC-A1 cells with BFA and blotted p53 and PrP expression. We found that BFA treatment reduced the level of p53 in BxPC-3 cells (Fig. 4A). However, BFA treatment of BxPC-3 cells significantly enhanced PrP expression (Fig. 4A). Although we did not detect p53 expression in H1299 and SPC-A1 cells, we detected enhanced PrP expression when those cells were treated with BFA for 24 h (Fig. 4B). Thus, BFA-induced PrP expression is independent of p53.

### BFA Induces Under-Glycosylated Cytosolic PrP

**Fig. 5:**
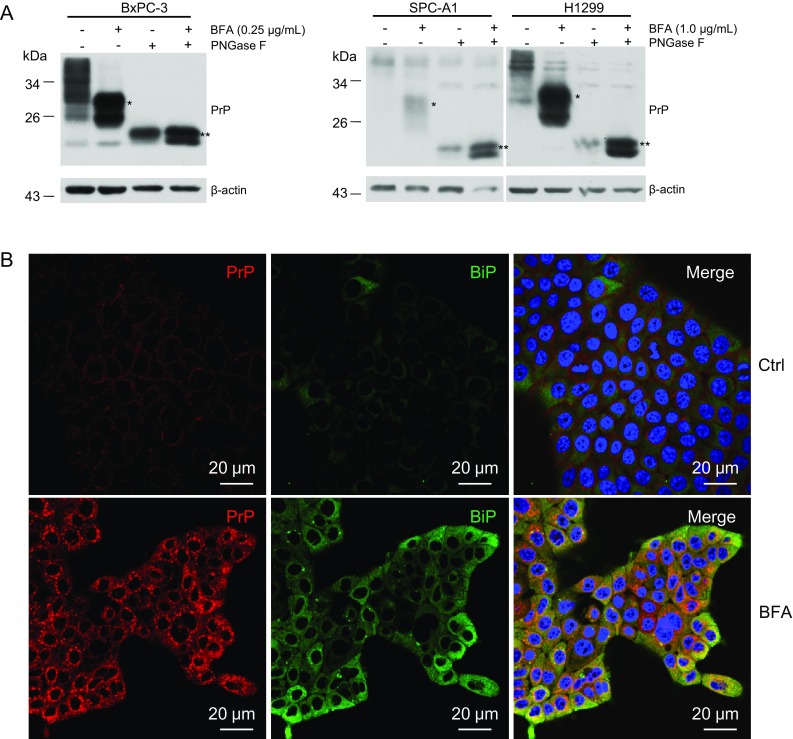
BFA treatment reduced PrP glycosylation leading to increased motility and accumulation of cytosolic PrP. **A** PNGase F treatment of BFA treated and non-treated cells reduced the apparent molecular weight of PrP. When comparing to non-BFA treated cells (Ctrl), PrP in BFA treated cells showed higher motility (indicated by *). After PNGase F treatment, the apparent molecular weight of PrP was further reduced (indicated by **). **B** Immunofluorescence staining of PrP (red) and BiP(green) showed that BFA treatment increased the levels of PrP and BiP, and the co-location of PrP and BiP.

PrP is normally a GPI-anchored glycoprotein localizing on the cell surface (Stahl *et al.*^1987^). It can be post translationally modified on either one of the two glycosylation sites or both glycosylation sites (Rogers *et al.*^1990^), and complex type *N*-linked glycans are added inside the Golgi apparatus (Lawson *et al.*^2005^). BFA blocks secretory proteins trafficking from ER to the Golgi apparatus. Thus PrP from BFA-treated cells shall not be modified with the complex type *N*-linked glycans. In fact, we observed an increased motility of PrP in BFA treated BxPC-3 and lung cancer cells (Fig. 2B). To confirm that those PrP from BFA treated PDAC cells are altered in *N*-linked glycosylation but are not due to other post translational modifications, we treated the cell lysates of BxPC-3, H1299, and SPC-A1 cells with PNGase F, which cleaves all types of asparagine-bound *N*-glycans as long as the oligosaccharide has the minimum length of the chitobiose core unit. We found that PNGase F-treated PrP migrated faster than the non-treated PrP (Fig. 5A). Thus, BFA treatment probably reduced complex type *N*-linked glycans of PrP. In addition, most PrP co-localized with BiP, a marker for ER (Fig. 5B), further confirming that PrP was retained inside the ER due to BFA treatment.

### BFA Treatment Enhances Apoptosis of *PRNP* Null BxPC-3 and SPC-A1 Cells

**Fig. 6:**
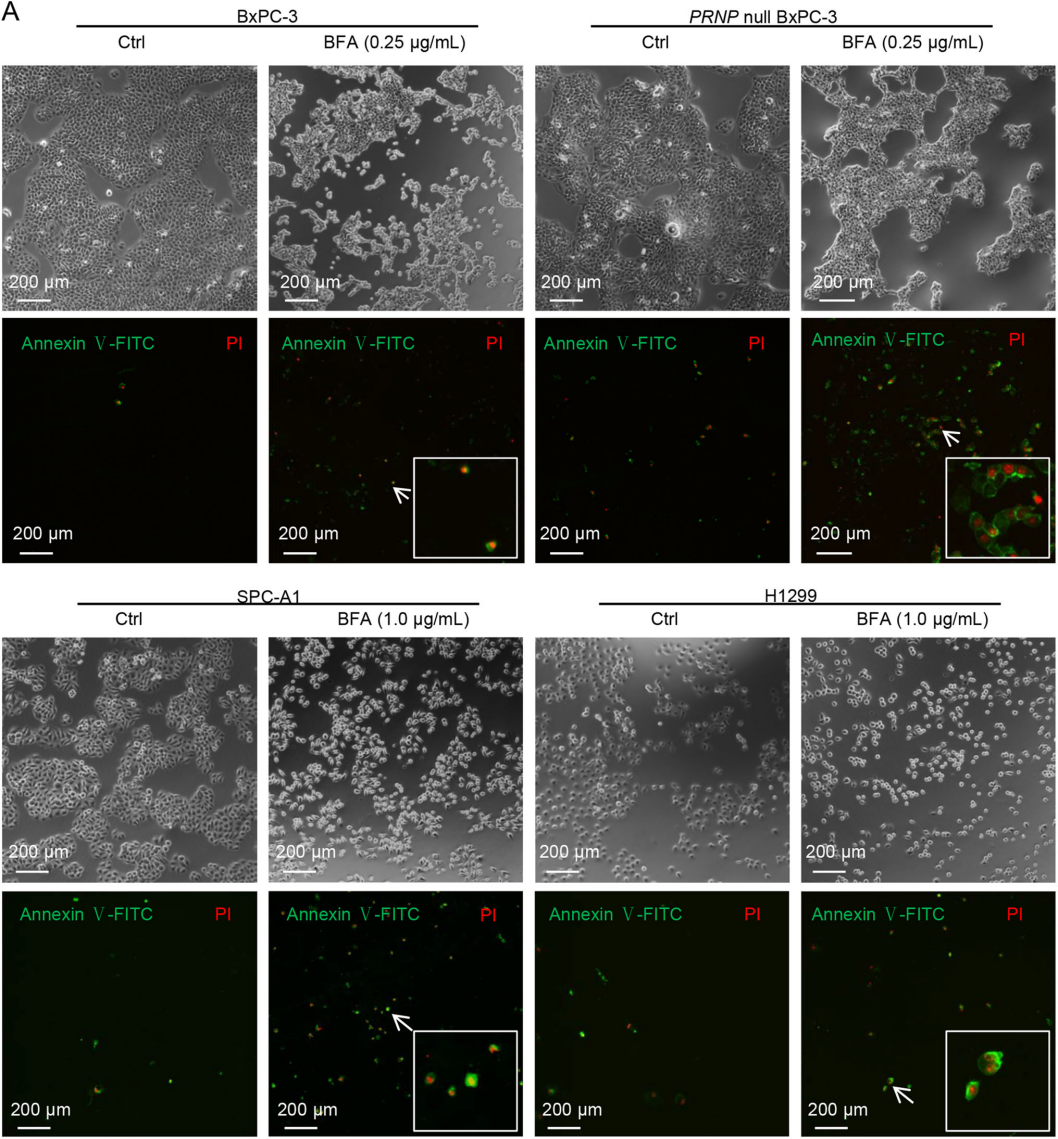

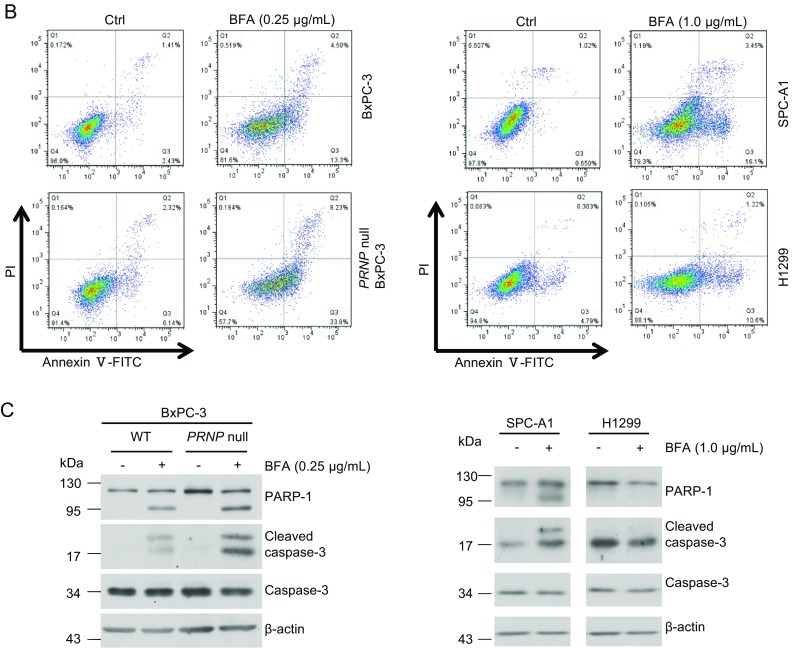
BFA treatment-induced cell apoptosis might depend on expression level of PrP. **A** Immunofluorescence staining showed that the level of PrP might affect BFA-induced cell apoptosis. BFA treatment induced more apoptosis for BxPC-3 cells compared to DMSO control treatment (Ctrl). More apoptosis was observed for *PRNP* null BxPC-3 cells than WT BxPC-3 cells when treated with BFA. BFA treatment induced significantly more apoptosis for SPC-A1 cells than control treatment. However, BFA treatment did not increase apoptosis of H1299 compared to DMSO control treatment. **B** Flow cytometry analysis showed that BFA treatment caused more apoptosis for SPC-A1 cells than for H1299 cells. Furthermore, *PRNP* null BxPC-3 cells had more apoptosis than PrP expressing BxPC-3 cells when treated with BFA. **C** Immunoblotting showed that BFA treatment resulted in more activated caspase-3 to cleave PARP-1. Furthermore, in *PRNP* null BxPC-3 cells, BFA treatment led to more activated caspase-3 and cleaved PARP-1.

Cytosolic PrP has been shown to cause cellular toxicity (Ma *et al.*^2002^; Rambold *et al.*^2006^; Wang *et al.*^2009^). In contrast, expression of PrP in many types of cancer cells prevents cell death (Diarra-Mehrpour *et al.*^2004^; Roucou *et al.*^2005^; Meslin *et al.*^2007^). Since we found that most PrP induced by BFA was cytosolic (Fig. 5B), we then investigated if BFA treatment could result in apoptosis and whether cytosolic PrP contributes to apoptosis. We performed immunofluorescence staining of *PRNP* null BxPC-3, PrP WT BxPC-3, SPC-A1, and H1299 cells treated with BFA for 24 h with propidiumiodide (PI) and Annexin V. Annexin V positive staining is for early apoptotic cells, whereas Annexin V and PI double positive staining are for late apoptotic cells. We found that BFA treatment significantly reduced the amount of *PRNP* null BxPC-3, WT PrP BxPC-3, and SPC-A1 cells in the petri dish (Fig. 6A), implying that those reduced cells were dead. However, we cannot exclude the possibility that BFA treatment may reduce cancer cell proliferation (Han *et al.*^2018^). On the contrary, BFA treatment did not significantly diminish the amount of H1299 cells that adhered to the petri dish (Fig. 6A), implying that H1299 cells were resistant to BFA treatment. In addition, *PRNP* null BxPC-3 cells had much more apoptosis than PrP expressing BxPC-3 cells which were induced to express high levels of cytosolic PrP (Fig. 6A). Accordingly, SPC-A1, which was stimulated to express low levels of PrP, also showed more apoptosis than H1299 cells, which expressed high levels of PrP (Fig. 6A). To quantify the percentage of cell death due to BFA treatment, we performed flow cytometry assays of SPC-A1, H1299, *PRNP* null BxPC-3, and WT PrP expressing BxPC-3 cells after 24 h of BFA treatment. We found that BFA treatment indeed caused significantly more SPC-A1 cell death than H1299 cells (Fig. 6B). In addition, BFA treatment resulted in significantly more *PRNP* null BxPC-3 cell death compared to PrP expressing BxPC-3 cells (Fig. 6B). Thus, ER stress-induced apoptosis may be dependent on the levels of PrP expression. Two of the best characterized apoptosis inducers are cleaved PARP-1 and caspase-3. We then blotted the cell lysates with antibodies against caspase-3 and PARP-1 and found that BFA treated PrP null BxPC-3 had more activated caspase-3 and cleaved PARP-1 than PrP expressing BxPC-3 cells (Fig. 6C). In addition, PrP high expressing H1299 showed decreased reaction to activated caspase-3 and cleaved PARP-1 than SPC-A1 cells, which express low level of PrP (Fig. 6C). These results were consistent with the apoptosis analysis and proved that PrP could protect cells from apoptosis induced by ER stress.

## Discussion

Herein, we provided evidence that the expression levels of PrP vary greatly among four human lung cancer cell lines, with A549 and H1299 having the highest levels, followed by H157 and then SPC-A1. The reason for this heterogenous pattern is not known. We posit that PrP contributes to lung cancer cell biology by engaging the UPR. ATF4, a pivotal UPR sensor was undetectable in untreated BxPC-3, SPC-A1, and H1299 cells. However, when treated with three different UPR-inducing agents, BFA, Thps or TM, there was a significant induction of ATF4 at transcriptional and translational levels. We found that of the three UPR inducers, BFA was by far the most potent inducer of ATF4 expression. Similar results have been observed when human neurons, astrocytes, and breast cancer MCF-7 cells were treated with BFA (Dery and LeBlanc 2017). Since BFA, but neither TM nor Thps, can transcribe and proteolytically activate luman, a non-canonical UPR transcription factor, which in turn transactivates PRNP expression (DenBoer *et al.*^2005^; Dery and LeBlanc 2017), it is likely that the stronger *PRNP* expression induced by BFA is mediated by luman. Interestingly, SPC-A1 cells, which expressed the lowest PrP, had a stronger ATF4 response compared to the high PrP expressing H1299 and BxPC-3 cells.

In the canonical PERK-eIF2α-ATF4 pathway, elevated p-eIF2α blocks translation of most genes but specifically enhances ATF4 translation (Harding *et al.*^2000^). eIF2α was constitutively expressed in the tested cell lines in the absence of UPR stimulants. However, when the cells were stimulated with UPR inducers, the levels of p-eIF2α were up-regulated in H1299 cells but were less obvious in SPC-A1 cells. Another UPR sensor, XPB-1s, is also constitutively expressed in the tested cell lines; treatment with UPR inducers does not modulate the expression levels of XPB-1.

The UPR down-stream sensor BiP is also significantly up-regulated in both cell types when stimulated with the ER stress inducers. Again, the PrP low-expressing cell line SPC-A1 cell responded significantly more than the PrP high-expressing cell line H1299 cell. It has been reported earlier that BiP is the chaperon protein, physically associated with PrP, guiding its folding, and thus, plays a role in maintaining the quality control in the maturation of PrP (Jin *et al.*^2000^). Higher levels of PrP in BxPC-3 and H1299 cells may have trapped BiP reducing their availability.

The strongest evidence suggesting that PrP plays a role in the UPR is our observation that the expression of PrP is up-regulated in mRNA and in protein levels when the two cell lines where treated with the UPR inducers. As expected, the PrP protein isoforms up-regulated were quite different among the three inducing agents. Unlike BFA which promoted the accumulation of the under-glycosylated PrP, TM preferentially up-regulated the expression of un-glycosylated PrP. The lowest molecular weight bands were un-glycosylated PrP because after treatment with PNGase F to remove the *N*-linked glycans, all PrP species collapsed into the lowest molecular species. Interestingly, the high PrP-expressing BxPC-3 and H1299 cells appeared to express even higher levels of PrP when treated with the UPR stimulants compared to the low-PrP expressing SPC-A1 cells.

When the two lung cancer cell lines were treated with BFA, there was an increase in apoptotic cells, suggesting the ER stress had increased cell death. Interestingly, the increase in apoptotic cells was more pronounced in low-PrP expressing SPC-A1 cells compared to the high-PrP expressing H1299 cells. In addition, *PRNP* null BxPC-3 cells have significantly more apoptosis than wild type PrP expression BxPC-3 cells. This observation is also consistent with the immunoblotting results showing that the levels of cleaved PARP-1 and activated caspase-3 were also higher in SPC-A1 cells or *PRNP* null BxPC-3 cells. Collectively, these findings add credence to our interpretation that PrP has a protective function in UPR and apoptotic cell dead. Persistent ER stress can cause cancer cell death (Clarke *et al.*^2014^). However, cancer cells also harness ER stress for their own benefits (Kaufman 1999). To take advantage of ER stress, cancer cells must implement mechanism(s) to prevent ER stress-induced cell death. This is probably one reason that the expression of PrP is up-regulated in many different human cancers.

In summary, we have provided strong evidence that the levels of PrP might contribute to cancer cells biology by engaging the UPR. Most importantly, cells with a lower PrP level have a higher level of UPR than cells with higher PrP levels. It will be important to further these studies with additional cell lines. Nonetheless, our study supports the hypothesis that the role PrP plays in tumor biology is cell-context dependent.

## Electronic supplementary material

Below is the link to the electronic supplementary material.

Supplementary material 1 (PDF 105 kb)
