# Supplementary material for: Prion Protein Protects Cancer Cells against Endoplasmic Reticulum Stress Induced Apoptosis
Source: Virol Sin. 2019 Apr 24;34(2):222–34. doi: 10.1007/s12250-019-00107-2 (PMC6513834; doi:10.1007/s12250-019-00107-2)
Supplement: Supplementary file 1 — Supplementary material 1 (PDF 105 kb) [file 12250_2019_107_MOESM1_ESM.pdf]

VIROLOGICA SINICA

Electronic Supplementary Material

Prion Protein Protects Cancer Cells against Endoplasmic Reticulum Stress Induced Apoptosis

Zhenxing Gao[1,#], Min Peng[2,#], Liang Chen[2], Xiaowen Yang[3], Huan Li[1], Run Shi[1], Guiru Wu[1], Lili Cai[1], Qibin Song[2], Chaoyang Li[1,✉]

1. State Key Laboratory of Virology, Wuhan Institute of Virology, Chinese Academy of Sciences, Wuhan 430071, China
2. Department of Oncology, Renmin Hospital of Wuhan University, Wuhan 430060, China
3. Department of the First Abdominal Surgery, Jiangxi Tumor Hospital, Nanchang 330029, China

Supporting information to DOI: 10.1007/s12250-00107-2

Table S1. The primers used for quantitative PCR

Primer names	Sequences of primers
PRNP-F	5'-GTGACTATGAGGACCGTTACTATC-3'
PRNP-R	5'-TGACCGTGTGCTGCTTGA-3'
ATF4-F	5'-TGAGCAGCGAGGTGTTGGT-3'
ATF4-R	5'-CCATCCACAGCCAGCCATTC-3'
XBP1-F	5'-ATGGATTCTGGCGGTATTGACT-3'
XBP1-R	5'-GGAGGCTGGTAAGGAACTGG-3'
β-ACTIN-F	5'-ATCGTGCGTGACATTAAGGAG-3'
β-ACTIN-R	5'-GGAAGGAAGGCTGGAAGAGT-3'
